# Development of Fe/SiBr/Si₃N₄/silica fume nanocomposites from recycled metal waste for industrial applications

**DOI:** 10.1038/s41598-024-81657-8

**Published:** 2025-01-09

**Authors:** Mohammed A. Taha, S. A. Gad, Rasha A. Youness

**Affiliations:** 1 Solid State Physics Department, National Research Centre, El Buhouth St., Dokki, Giza, 12622 Egypt; 2 Pharos University in Alexandria, Canal Mahmoudiah Street, Smouha, Alexandria Egypt; 3 Spectroscopy Department, National Research Centre, El Buhouth St., Dokki, Giza, 12622 Egypt

**Keywords:** Recycled metal waste, Tribo-mechanical properties, Electrical properties, Magnetic properties, Industrial applications, Nanoscale materials, Structural materials

## Abstract

Due to the high cost of raw materials, this work aims to benefit from metal waste, especially iron (Fe) and silicon bronze, which results from turning workshops and recycling them to obtain nanocomposites for industrial applications. In this respect, Fe/SiBr/Si_3_N_4_/silica fume nanocomposites possessing superior mechanical, wear, and magnetic characteristics have been produced using powder metallurgy (PM) technology. Milled sample particle size, crystal size, and phase composition were investigated using X-ray diffraction (XRD) technique and transmission electron microscopy (TEM). The powders were compressed and sintered in argon to get excellent sinterability. The sintered nanocomposites’ physical, mechanical, wear, electrical, and magnetic properties were investigated. The microstructure was also examined using field emission scanning electron microscopy (FESEM). The results showed a noticeable decrease in the size of particles and crystallite size after adding reinforcements, reaching 22 nm for the sample improved with 5 vol% silica fume and 5 vol% Si_3_N_4_ (FS4). In addition, after adding reinforcements, there was a clear improvement in the microhardness, Young’s modulus, and wear rate of Fe-SiBr, reaching 58, 27.89, and 43.21% percent for the sample FS4. Adding reinforcements harms the electrical conductivity of Fe-SiBr, as it decreases to 8.64 × 10^6^ S/m for the same previous sample. Finally, adding reinforcements slightly affects the decrease in magnetization of the nanocomposites.

## Introduction

The recycling of metals is essential for environmental sustainability and for obtaining raw resources. Metals like iron (Fe), copper (Cu), and their alloys are non-renewable resources, and their extraction from ores is energy-intensive, resulting in considerable greenhouse gas emissions, habitat damage, and water contamination. Recycling these metals reduces the need for mining, preserving natural landscapes, and reducing energy use^1–3^. Recycling Cu and its alloy requires up to 85% less energy than extracting and processing fresh copper from ore. Recycling iron conserves around 60–70% of the energy used in basic steel manufacture. Furthermore, recycling satisfies industrial demand by ensuring a consistent supply of raw materials. Through recycling, companies may diminish expenses, decrease their carbon footprint, and foster a circular economy where items are repurposed instead of disposed of, thus promoting sustained environmental well-being^4–7^. Fe and its alloys have a wide range of applications in various industries, such as the automotive industry, brake discs, and engine parts, and are suitable for turbine blades and structural components. They are also used in cutting tools, wear-resistant parts, and magnetic applications, such as transformer cores, where their soft magnetic properties improve efficiency^8–10^. Despite the previous applications, Fe and its alloys have some fundamental defects that limit their effectiveness in the applications above. This encompasses inadequate wear resistance, restricted hardness, and vulnerability to corrosion and oxidation. Such faults may result in material deterioration, particularly in high-friction or corrosive settings^11,12^. It is essential to acknowledge that copper and its alloys are mostly favored due to their enhancement of thermal conductivity in frictional contact and their ability to sustain the coefficient of friction at elevated temperatures^13,14^. Furthermore, incorporating Cu presents an economic advantage by lowering the temperature necessary for sintering during the preparation process. This may be because the melting temperature of copper (~ 1080 °C) is lower than that of Fe (~ 1538 °C)^13,15^. In this regard, ceramics such as aluminium oxide (Al₂O₃)^16^, zirconium oxides (ZrO_2_)^17^, silicon carbide (SiC)^18^, and boron carbide^19^ are included to enhance attributes like wear resistance, hardness, and corrosion resistance.

It is worth noting that adding a single ceramic may not improve the required properties, so we resort to using a hybrid ceramic^20,21^. Silicon nitride (Si₃N₄) is a high-performance ceramic distinguished by its remarkable mechanical strength, thermal stability, and resistance to wear and corrosion. Its significance arises from its capacity to retain strength at high temperatures^22^. As an economical ceramic addition in metal matrices, Si₃N₄ markedly enhances toughness, wear resistance, and thermal performance, particularly in applications such as cutting tools, engine components, and bearings. These advances allow metal composites to excel in adverse environments, prolonging their durability and enhancing efficiency in industrial operations^23^. It is recommended to use ceramic waste materials such as fly ash, silica fume, granite, activated carbon, and other similar materials. This is because the choice of composite components significantly impacts the cost^24^. There are various methods for preparing metal-based composites, and one of these methods is powder metallurgy, as this method uses sintering and compaction to create components out of powdered materials. Because it gives fine control over the composition and microstructure of materials, it is essential for the preparation of composites^25–27^. With this method, metals and ceramics may be combined to create metal or ceramic matrix composites, which have higher strength, wear resistance, and thermal stability, among other features. Powder metallurgy has several benefits, including less material waste, the capacity to form intricate structures, and the ability to deal with materials that are difficult to treat using conventional techniques^28,29^. One of the most important elements of development in the industry is the low cost of the raw materials used, and the most important of these materials is iron due to its use in the various industrial applications mentioned previously. Therefore, it is necessary to have it available in large quantities and at a lower cost.

The novelty in this manuscript is the recycling of Fe and SiBr waste resulting from lathe workshops and using it as a matrix and improving it with silica fume waste and Si_3_N_4_ nanoparticles, whether all ceramics individually or a hybrid between them. Conversely, using the powder metallurgy technique, we can enhance the nanocomposites’ wear resistance and mechanical characteristics, such as microhardness, compressive strength, and elastic moduli, while maintaining their magnetic properties. Additionally, we can investigate their electrical conductivity for their potential use in diverse industrial applications.

## Materials and methods

### Samples preparation

**Fig. 1:**
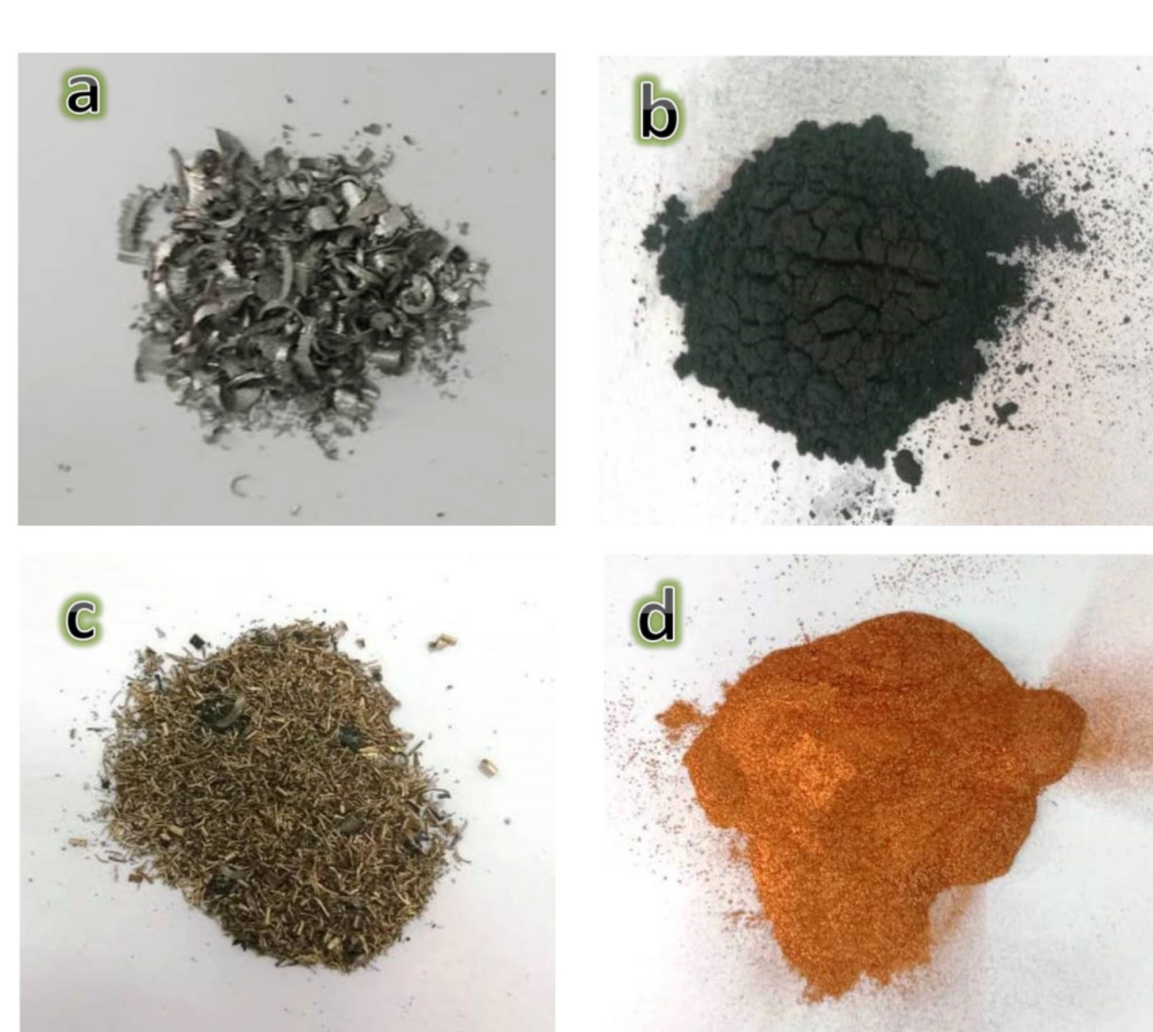
Photographs of (**a**) Fe waste before milling, (**b**) Fe waste after milling, (**c**) SiBr waste before milling, and (**d**) SiBr waste after milling.

The Fe and SiBr waste from lathe workshops was first fragmented into small pieces a few millimeters in width and then processed into waste powders through high-energy ball milling for four hours. Photographs of Fe and SiBr waste taken before and after milling are depicted in Fig. 1a–d. We are using Si_3_N_4_, which has a purity of 99.95 and a particle size of 25 nm, and silica fume, which has a purity of 99.9 and a particle size range of 38.9 to 81.8 nm, as a reinforcer. Firstly, the base matrix of this work was Fe 15vol.% SiBr is mixed for 25 h with a ball-to-powder ratio (BPR) equal to 5:1 and a speed of 150 rpm. Secondary, varied volume percentages of the mono and hybrid (Si_3_N_4_ and silica fume) were introduced to the Fe-SiBr base. The composition of Fe, SB, and silica fume waste powders is tabulated in Tables 1, 2 and 3, respectively. The batch compositions designed for nanocomposites, with their abbreviations, are tabulated in Table 4. Each sample powder was milled for 25 h at 450 rpm with a BPR of 20:1. The milled powders were pressed with a hydraulic machine with a pressure of 40 MPa and then sintered in an argon atmosphere for one hour at 1100 °C with a heating rate of 5 °C/min.

**Table 1:** Composition of Fe waste powders (wt%).

Element	Fe	C	Mn	Al	*P*	Others
wt%	99.72	0.06	0.05	0.06	0.03	0.08

**Table 2:** Composition of SB waste powders (wt%).

Element	Cu	Si	Zn	Mg	Fe
wt%	94.05	3.10	1.72	0.12	1.11

**Table 3:** Composition of silica fume waste powder ( wt%).

Element	SiO_2_	Al_2_O_3_	Fe_2_O_3_	CaO	MgO	K_2_O	Na_2_O	LOI
wt%	94.41	0.80	2.23	0.49	1.13	0.06	0.06	1.26

**Table 4:** Batch design of the investigated prepared nanocomposites.

Sample name	Composition (Vol.%)		
	Fe-15 SiBr	Si_3_N_4_	Silica fume
FS0	100	–	–
FS1	95	–	5
FS2	95	5	–
FS3	95	2.5	2.5
FS4	90	5	5

### Characterization of the prepared powders

X-ray diffraction (XRD; Philips PW) technique was used to detect the phases of the milled powders. X-ray line broadening for the principle (*h k l*) planes (1 1 1, 211, and 2 2 0) was used to calculate the crystalline size (D), lattice strain (ε), and dislocation density (δ) were determined using the formulae described in Ref^21^

1 $$\:\text{D}=\:\frac{0.9{\uplambda\:}}{\text{B}\text{c}\text{o}\text{s}{\uptheta\:}}$$

2 $$\:{\upvarepsilon\:}=\:\frac{\text{B}}{4\text{tan}{\uptheta\:}}$$

3 $$\:{\updelta\:}=\:\frac{1}{{D}^{2}}$$

where k (wavelength) = 1.54059 A (Cu–Ni radiation), B is the full width at half maximum, and θ is the angle in radians. High-resolution transmission electron microscopy (HRTEM; JEOL–JEM2100) was used to investigate the particle size of the reinforcements and milled powder sample.

### Sinterability of the prepared nanocomposites

The milled powders were compacted into small compacts of desired sizes using the hydraulic pressing machine at room temperature. Samples of 15 mm diameter and 4 mm height were pressed in a hardened steel die at 30 MPa. Then, the compacted samples were sintered at 1100 °C in argon for one hour with a heating rate of 5 C/min. FESEM coupled with energy dispersive X-ray analysis **(**EDX) (type Quanta FEG250) was also used to examine the microstructure of sintered nanocomposite samples. The liquid displacement method measured the bulk density, relative density, and apparent porosity of each sintered sample^30^.

As mentioned in recent work, the microhardness of the sintered samples was measured by a Vickers tester according to ASTM: B933-09 with an applied load of 1.9 N for 10 s^31^.

4 $$\:Hv=1.854\frac{p}{{d}^{2}}$$

Where Hv is microhardness, P is applied load (1.9 N), and d is the indentation diagonal. The compressive strength of sintered samples was measured according to ASTM E9.

The longitudinal (V_L_) and shear (V_S_) velocities were measured using the pulse-echo technique. The Lame’s constants (i.e. λ and µ) were calculated using the bulk density of the sintered specimens according to the formula^32^:

5 $$\:{\uplambda\:}={\uprho\:}({V}_{L}^{2}-2{\text{V}}_{S}^{2})$$

6 $$\:{\upmu\:}\:={\uprho\:}{V}_{S}^{2}\:$$

The longitudinal modulus, Young’s modulus, shear modulus, bulk modulus and Poisson’s ratio (i.e. L, E, B, and G, respectively) of the sintered specimens were calculated according to the formula^33,34^:

7 $$\:L=\lambda\:+2\mu\:$$

8 $$\:G=\mu\:$$

9 $$\:E=\mu\:\frac{3\lambda\:+2\mu\:}{\lambda\:+\mu\:}$$

10 $$\:B=\lambda\:+\frac{2}{3}\mu\:$$

Dry sliding wear tests were performed on an air-on-disk wear tester according to ASTM G99 under sliding conditions using a pin-on-disc wear-testing apparatus. The process parameters of the wear test involved a speed of 0.8 m/s, a sliding time of 10 min, and applied loads of 30 N. The wear rate (W) of sintered samples is calculated by the following^35^:

11 $$\:\:\text{w}\text{e}\text{i}\text{g}\text{h}\text{t}\:\text{l}\text{o}\text{s}\text{s}=\text{w}\text{e}\text{i}\text{g}\text{h}\text{t}\:\text{b}\text{e}\text{f}\text{o}\text{r}\text{e}\:\text{w}\text{e}\text{a}\text{r}-\text{w}\text{e}\text{i}\text{g}\text{h}\text{t}\:\text{a}\text{f}\text{t}\text{e}\text{r}\:\text{w}\text{e}\text{a}\text{r}$$

12 $$\:\text{w}\text{e}\text{a}\text{r}\:\text{r}\text{a}\text{t}\text{e}=\frac{\text{w}\text{e}\text{i}\text{g}\text{h}\text{t}\:\text{l}\text{o}\text{s}\text{s}}{\text{S}\text{l}\text{i}\text{d}\text{i}\text{n}\text{g}\:\text{t}\text{i}\text{m}\text{e}}$$

A vibrating sample magnetometer (VSM; Lake Shore-7410-USA) was used to investigate the magnetic characteristics of all synthesized samples. The electrical conductivity (σ) of the sintered sample was measured at room temperature using the Keithley system according to the formula^30^:

13 $$\:{\upsigma\:}=\:\frac{\text{h}}{\text{R}\text{A}}$$

Where R, h, and A are the electrical resistance, the sample diameter, and the specimen’s surface area.

It is very important to note that the bulk density, relative density, apparent porosity, microhardness, ultrasonic velocities, compressive tests, electrical conductivity, and wear tests were measured four times to ensure the reproducibility of the readings.

## Results and discussion

### Characterization of the raw and prepared powders

#### Phase composition of the prepared powders

**Fig. 2:**
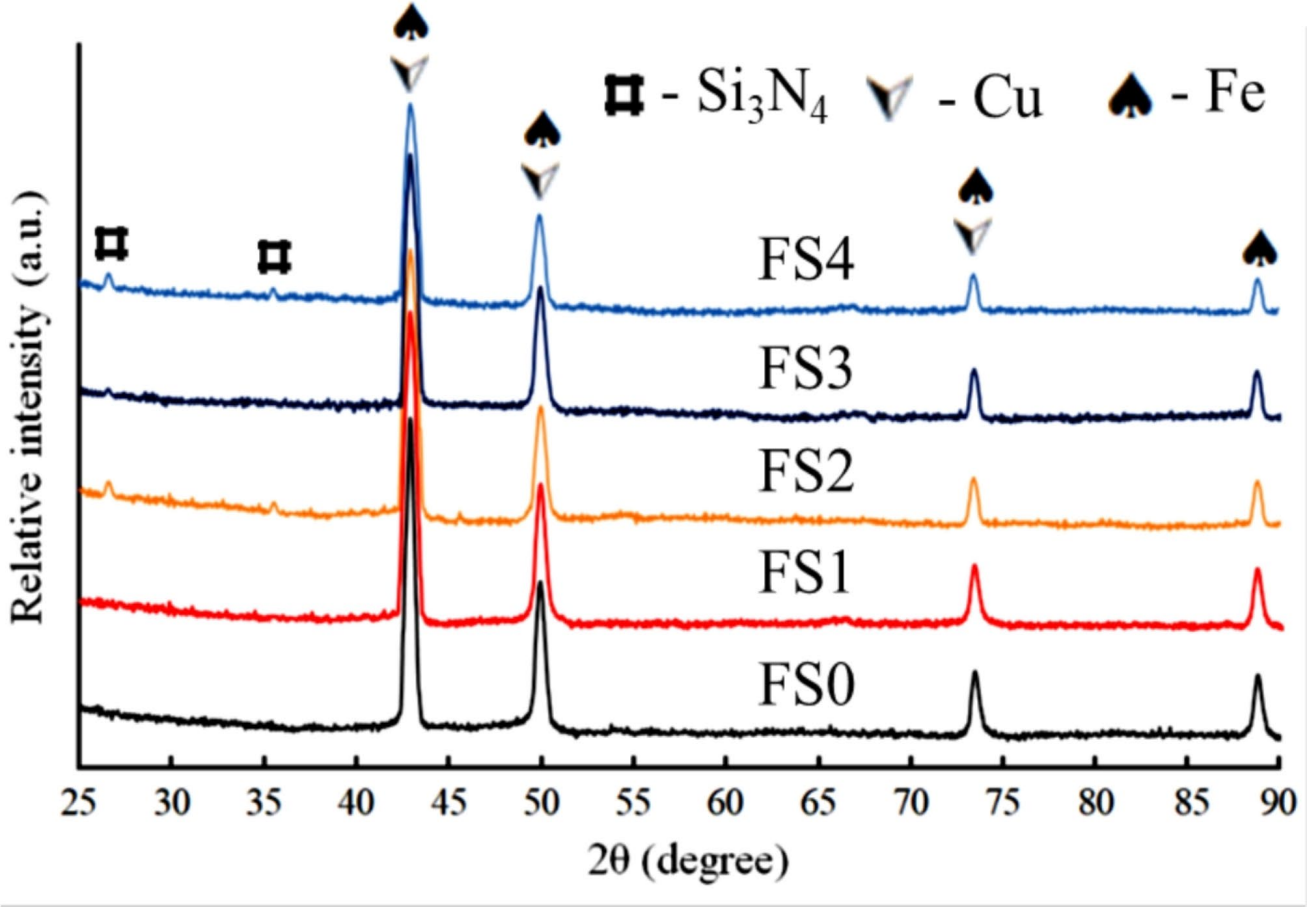
The XRD patterns of the FS00, FS1, FS2, FS3, and FS4 powders after 25 h of milling.

**Fig. 3:**
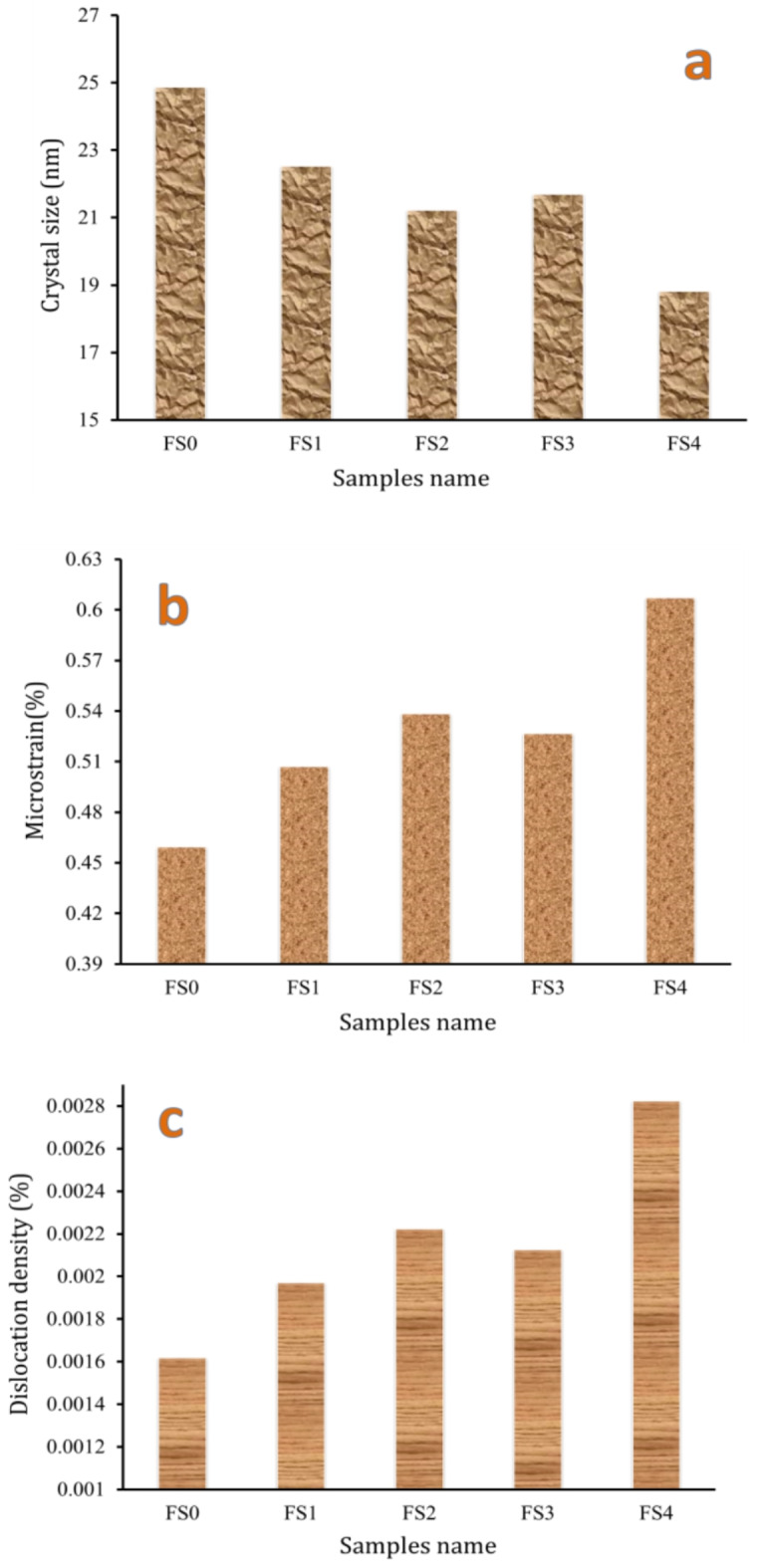
(**a**) Crystallite size, (**b**) lattice strain, and (**c**) dislocation density of milled powders.

Figure 2 displays the XRD patterns of Fe-SB-based nanocomposite powders reinforced with mono and hybrid of Si_3_N_4_ and silica fume after 25 h of milling. The XRD patterns indicate the presence of peaks corresponding to Fe, Cu, and Si_3_N_4_, as per ICCD file cards 89-4185 and 89-3830, respectively. Conversely, silica fume is amorphous, as shown by the lack of discernible diffraction peaks indicative of lattice periodicity^36^. A broad peak typical of amorphous silica is seen for two theta values between 15 and 25. According to the XRD cards, the Fe, Cu, and Si powder exhibits a cubic crystal structure, while Si_3_N_4_ exhibits a hexagonal crystal structure. Notably, since the other elements in the Fe and SB are present in tiny quantities, their typical peaks cannot be seen in the XRD chart. Finally, The Si_3_N_4_ appeared at 2*θ* = 26.683° and 35.547°, corresponding to (2 0 0) and (2 1 0). After the milling process, a significant broadening with a decrease in the intensity of peaks of the Fe-SB base after adding ceramic reinforcements. To describe the microstructure of the synthesized nanopowders, the crystallite size, dislocation density, and microstrain of the milled powders were determined by the analysis of the broadening of their diffraction peaks shown in Fig. 3(a-c). In addition, as the mono and hybrid ceramics were added, the crystal size decreased. At the same time, dislocation density and microstrain escalated owing to intense plastic deformation and grain size refinement during the milling process caused by the incorporation of nano-reinforcement particles^37^.

#### Morphology of the prepared powders

**Fig. 4:**
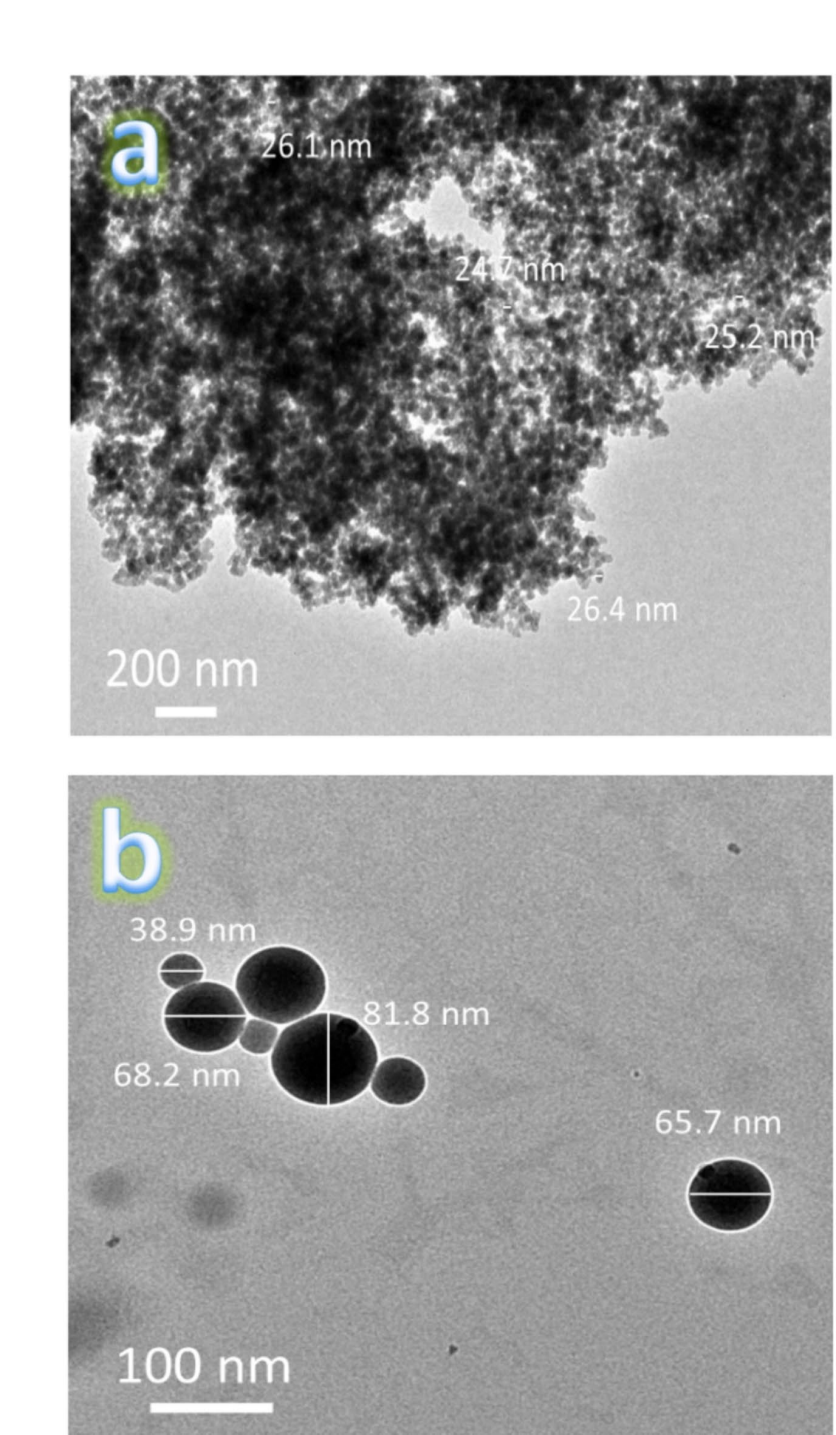
HRTEM micrographs of used ceramic reinforcements, i.e., (**a**) Si_3_N_4_ and (**b**) silica fume nanoparticles.

**Fig. 5:**
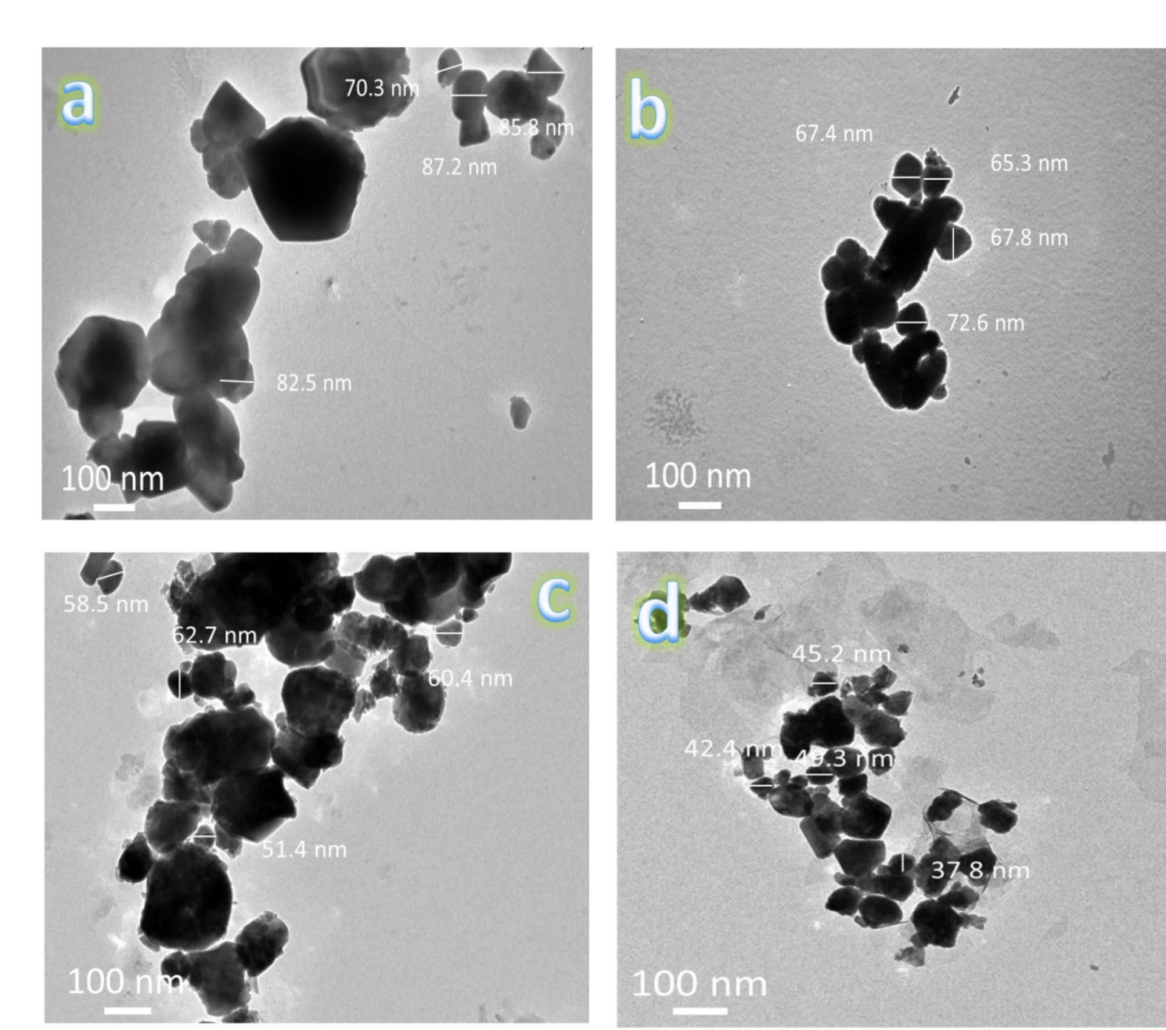
TEM micrographs of (**a**) FS0, (**b**) FS1, (**c**) FS2, and (**d**) FS4 milled powders.

**Fig. 6:**
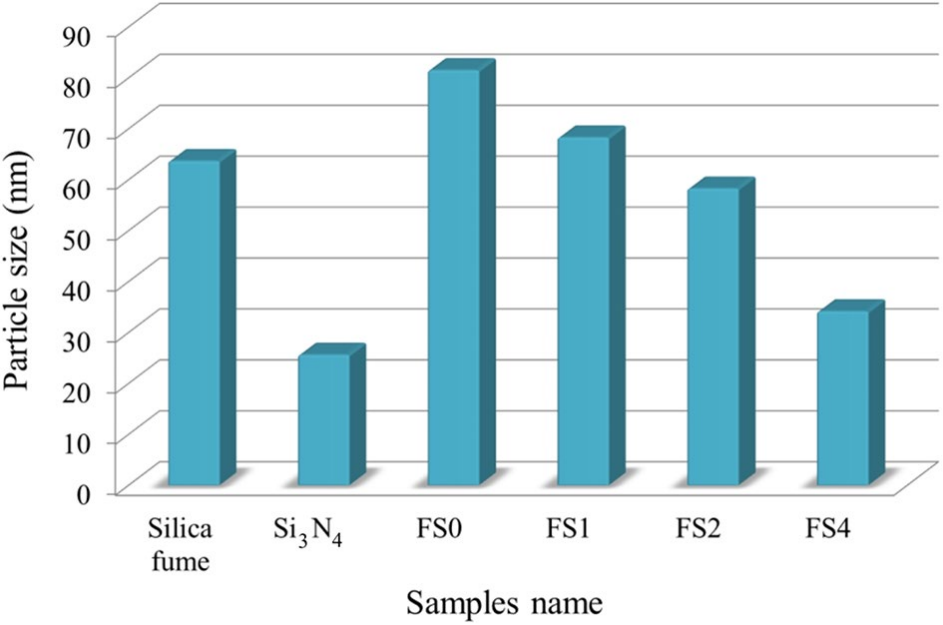
Effect of added silica fume and/or Si_3_N_4_ reinforcements on the particle size of milled powders.

Figure 4a, b shows the HRTEM photos of Si_3_N_4_ and silica fume nanoparticles. The reinforcement powders appear as particles with agglomerates and sizes 25.61 and 63.65 nm, respectively. TEM images of FS0, SF1, FS2, and FS4 powdered after milling for 25 h are shown in Fig. 5(a-d), respectively. The FS0 sample aggregates together, as illustrated in Fig. 5a, due to its 81.48 nm particle size and flexibility. In contrast, after adding mono reinforcement (silica fume and Si_3_N_4_), the agglomeration has been somewhat reduced, and the sizes of the particles are 68.28 and 58.25 nm, respectively, as shown in Fig. 5b and c. By adding hybrid reinforcement (5 vol% silica fume + 5 vol% Si_3_N_4_), we notice a further decrease in the aggregation of particles, with the particle size decreasing to 34.14 nm. Generally, the metal particles (Fe and SiBr) suffer from deformation, and the ceramics (silica fume and Si_3_N_4_) suffer fragmentation. Therefore, the metal particles start to weld at the beginning of the milling process, while the ceramics reinforcement ones come between two or more metal particles. This implies that the ceramic particles linger at the interfacial boundaries of the welded metal particles, resulting in nanocomposite powders with smaller particle sizes^38^. To a reasonable extent, these results agree with those reported in Refs^3,30,39^. Figure 6 shows the effect of added mono and hybrid ceramic particles on the particle size of the Fe-SiBr matrix.

### Characterization and properties of the sintered samples

#### Microstructure

**Fig. 7:**
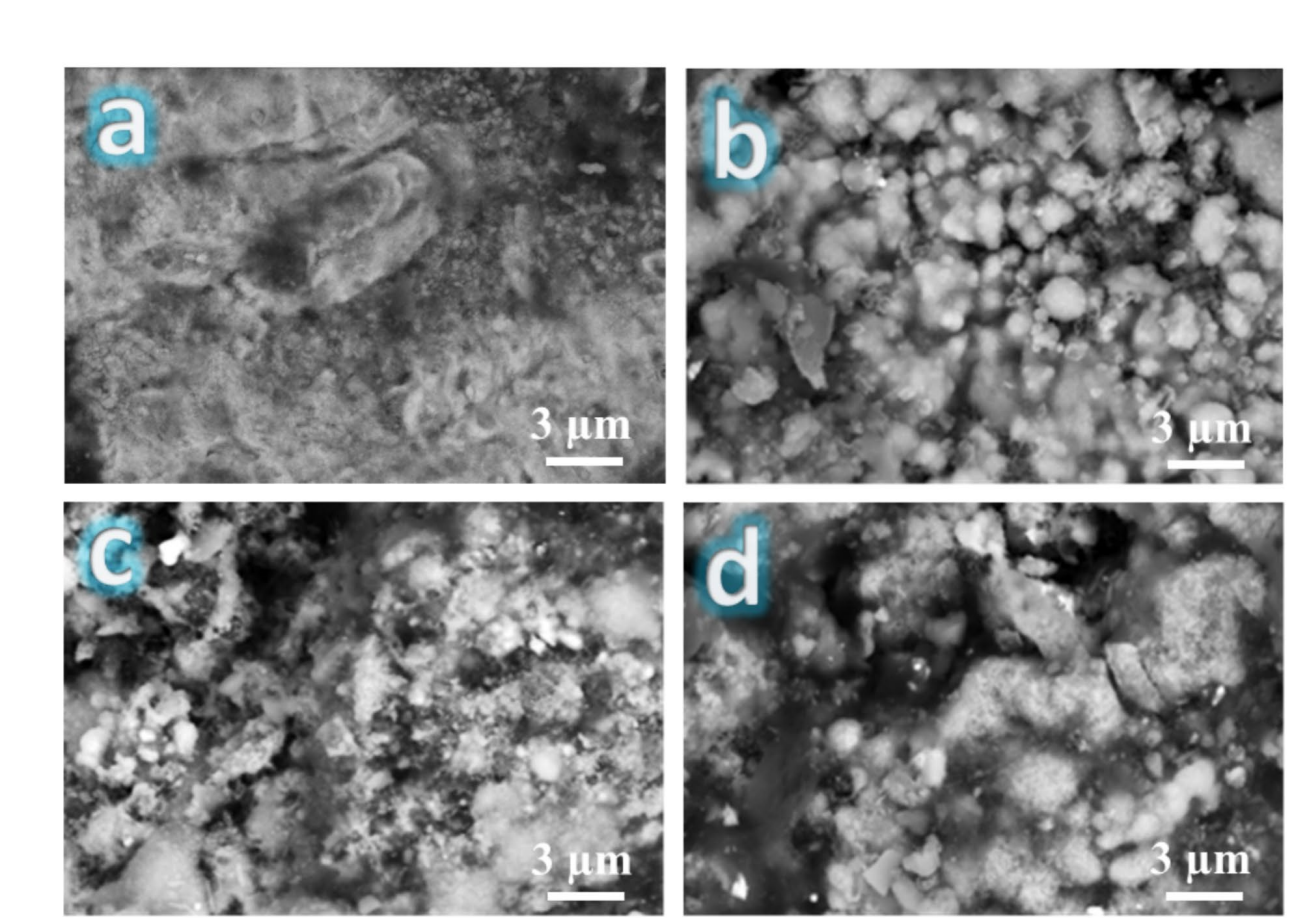
SEM images of (**a**) FS0, (**b**) FS1, (**c**) FS2, and (**d**) FS4 after sintered at 1100 °C for one hour in argon.

**Fig. 8:**
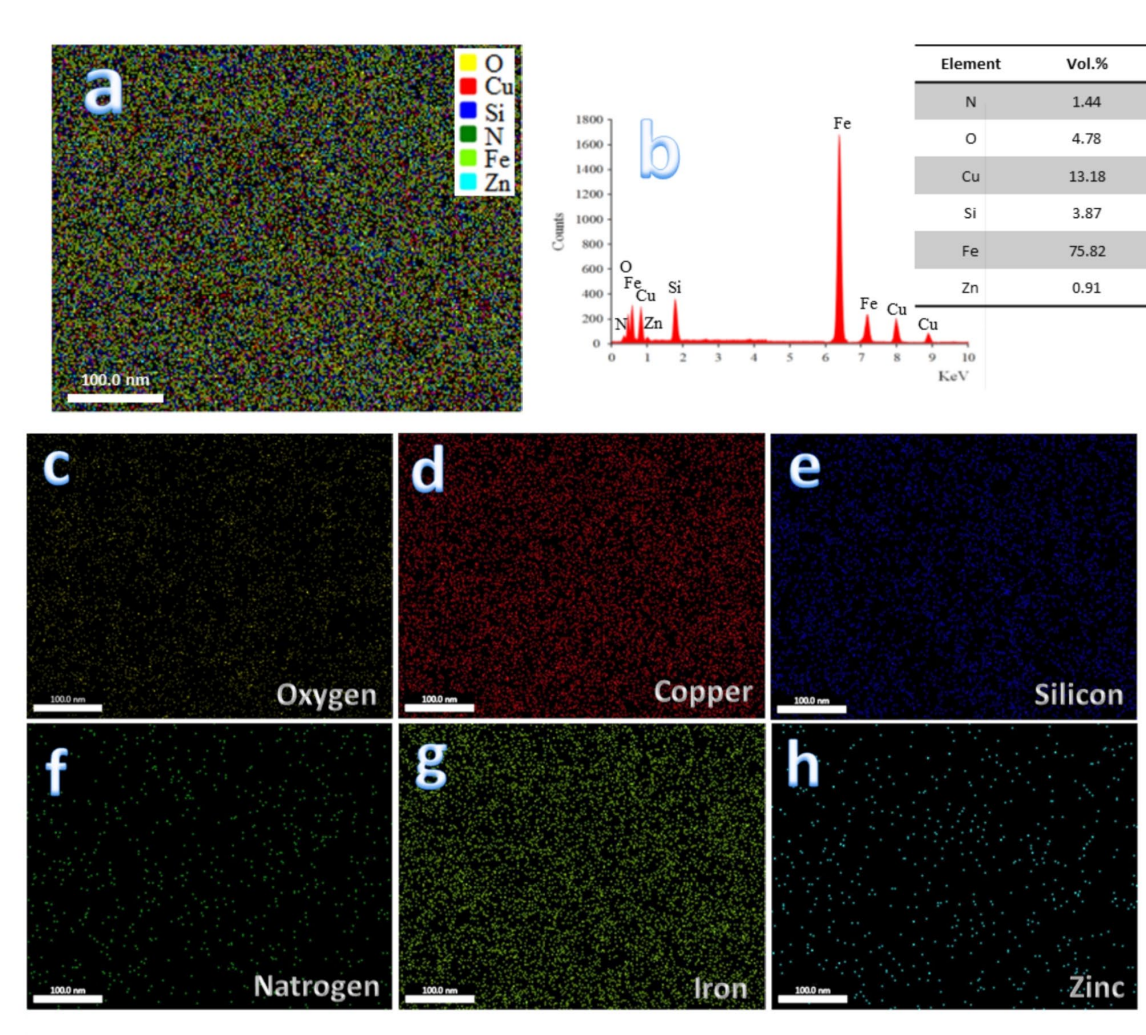
(**a**) EDX mapping of all constituents of FS4 sample, (**b**) EDX spectra and elemental mapping of the constituents forming, i.e., (**c**) oxygen, (**d**) copper, (**e**) silicon, (**f**) nitrogen, (**g**) iron, and (**h**) zinc.

Figure 7 displays the SEM micrographs of SF0, SF1, SF2, and FS4, nanocomposite specimens, sintered at 1100 °C for one hour in argon. Generally, it is possible to see that the unreinforced sample (FS0) shows good densification. Still, after adding the mono reinforcement, such as silica fume (FS1) or Si_3_N_4_ (FS2), the densification decreases and continuously decreases for samples containing hybrid reinforcements (FS4). Notably, it was also shown that the porosity of samples increased as the reinforcing particles were added. However, good densification behavior is produced due to choosing an appropriate sintering temperature, which promotes diffusion throughout the heating phase. Figure 8 illustrates the EDX spectrum and the distribution mapping of each component in the FS4 sample. The data indicates that no additional components are present, eliminating contamination during the milling or sintering operations. Another conclusion derived from this image is that a uniformly homogenous distribution of reinforcement nanoparticles was achieved in the Fe-SiBr matrix.

#### Physical properties

**Fig. 9:**
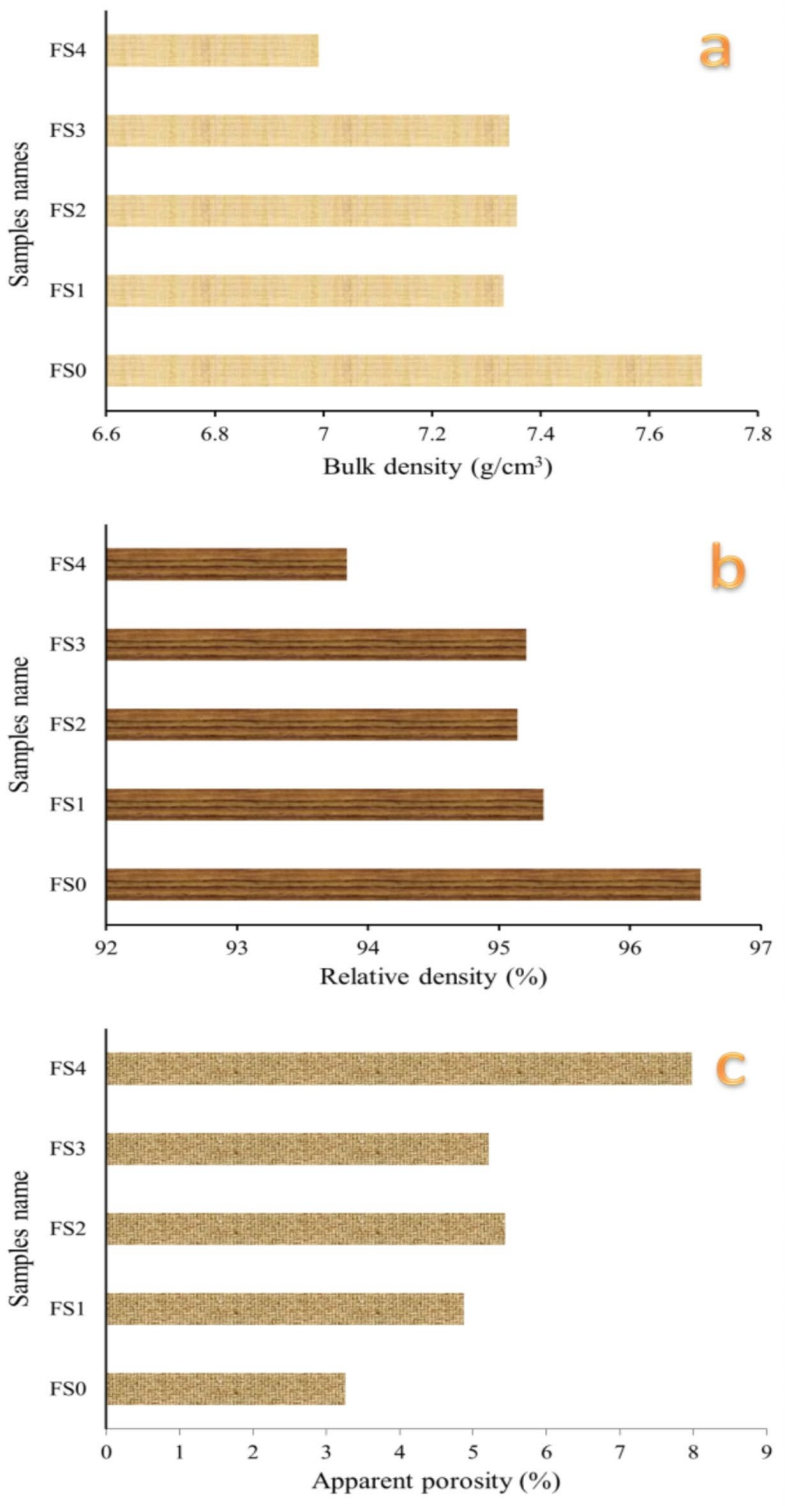
(**a**) Bulk density, (**b**) relative density, and (**c**) apparent porosity of the prepared nanocomposites.

Figure 9a–c illustrates the impact of ceramic reinforcement on the bulk density, relative density, and apparent porosity, respectively, of Fe-SiBr-based nanocomposites sintered for one hour at 1100 °C in an argon environment. According to the mixture rule, the theoretical densities of FS0, FS1, FS2, FS3, and FS4 samples are 7.972, 7.690, 7.732, 7.110, and 7.450 g/cm^3^, respectively. The figure clearly illustrates that the specimens’ bulk and relative densities significantly decreased with an increase in the reinforcement volume percent particles. Conversely, the apparent porosity increased by the same factor. The relative density of previous samples is 96.54, 95.34, 95.14, 95.21, and 93.84%, respectively, while apparent porosity is 3.26, 4.87, 5.44, 5.21and 7.89, respectively. This decrease in densities and increase in apparent porosity of sintered Fe-SB base as the mono and hybrid reinforcements may be due to the density of Si_3_N_4_ and silica fume ( 3.2 and 2.2 g/cm^3^, respectively) lower than Fe and Cu (7.87 and 8.96, respectively). The significant disparity in melting temperatures between the base components (Fe and SB ~ 1538 and 1.025 °C, respectively) and the reinforcing materials (Si_3_N_4_ and silica fume ~ 1710 and 2200 °C, respectively) results in reduced particle rearrangement during sintering. Moreover, the density of reinforcements is inferior to that of the metal base.

#### Mechanical properties

**Fig. 10:**
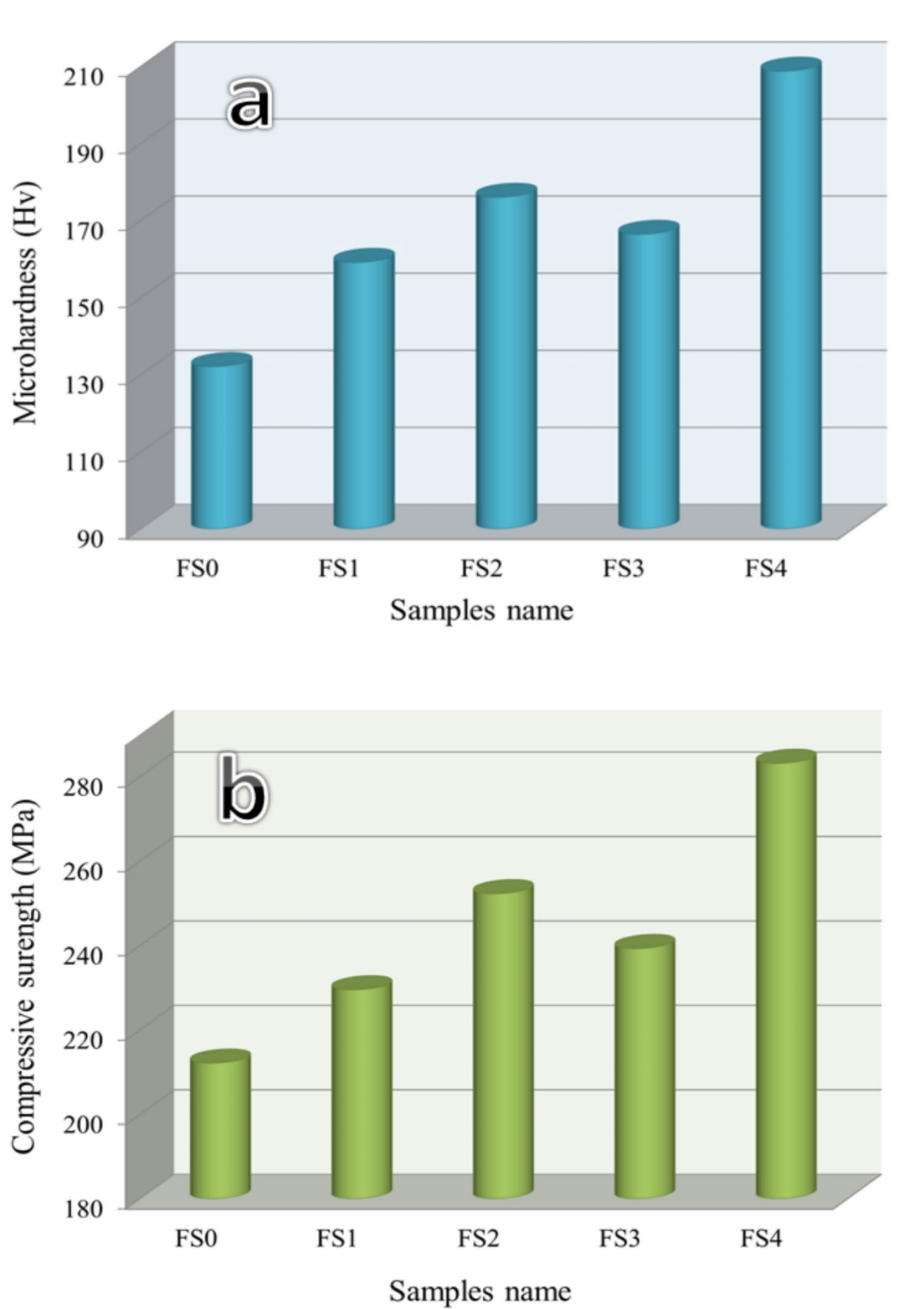
(**a**) Microhardness, and (**b**) compressive strength of Fe-SiBr based and its nanocomposites.

Figure 10a, b shows that the sintered Fe-SiBr base and nanocomposite samples exhibited average microhardness and compressive strength values. The findings show that adding Si_3_N_4_ or silica fume particles significantly raises the microhardness of the Fe-SB base. The microhardness of the Fe-SB base increases from 132.11 to 159.14, 176.01, 166.42, and 208.74 Hv due to the addition of 5vol.% silica fume, 5vol.% Si_3_N_4_, 2.5vol.% silica fume + 2.5vol.% Si_3_N_4_ and 5 vol% silica fume + 5 vol% Si_3_N_4_ reinforcement particles improved by about 20.46, 33.23, 25.97, and 58.01%, respectively. Generally, the enhanced microhardness of nanocomposite after added mono and hybrid reinforcement can be attributed to several factors. Firstly, the homogenous distribution of mono and hybrid reinforcements in the Fe-SB base secondarily reduced grain sizes of the base with added reinforcements, and finally, the existence of hard reinforcement particles (i.e., Si_3_N_4_ and silica fume)^40,41^. The following Eq. (14) helps to estimate this stiffness improvement better^42^:

14 $$\:H\text{v}=\:{\text{H}}_{\text{b}\text{a}\text{s}\text{e}}{\text{F}}_{\text{b}\text{a}\text{s}\text{e}}+{\text{H}}_{\text{S}\text{i}3\text{N}4}{\text{F}}_{\text{S}\text{i}3\text{N}4}+{\text{H}}_{\text{S}\text{i}\text{l}\text{i}\text{c}\text{a}\:\text{f}\text{u}\text{m}\text{e}}{\text{F}}_{\text{S}\text{i}\text{l}\text{i}\text{c}\text{a}\:\text{f}\text{u}\text{m}\text{e}}\:$$

The compressive strength of nanocomposites is the same trend as that of microhardness, which increases with added reinforcements. The base sample’s compressive strength is 212.14 MPa, and it increases to 229.51, 252.28, 239.27, and 283.15 MPa, resulting in improvements of approximately 8.19, 18.92, 12.79, and 33.47 MPa, respectively, after adding previous mono and hybrid reinforcements.

**Fig. 11:**
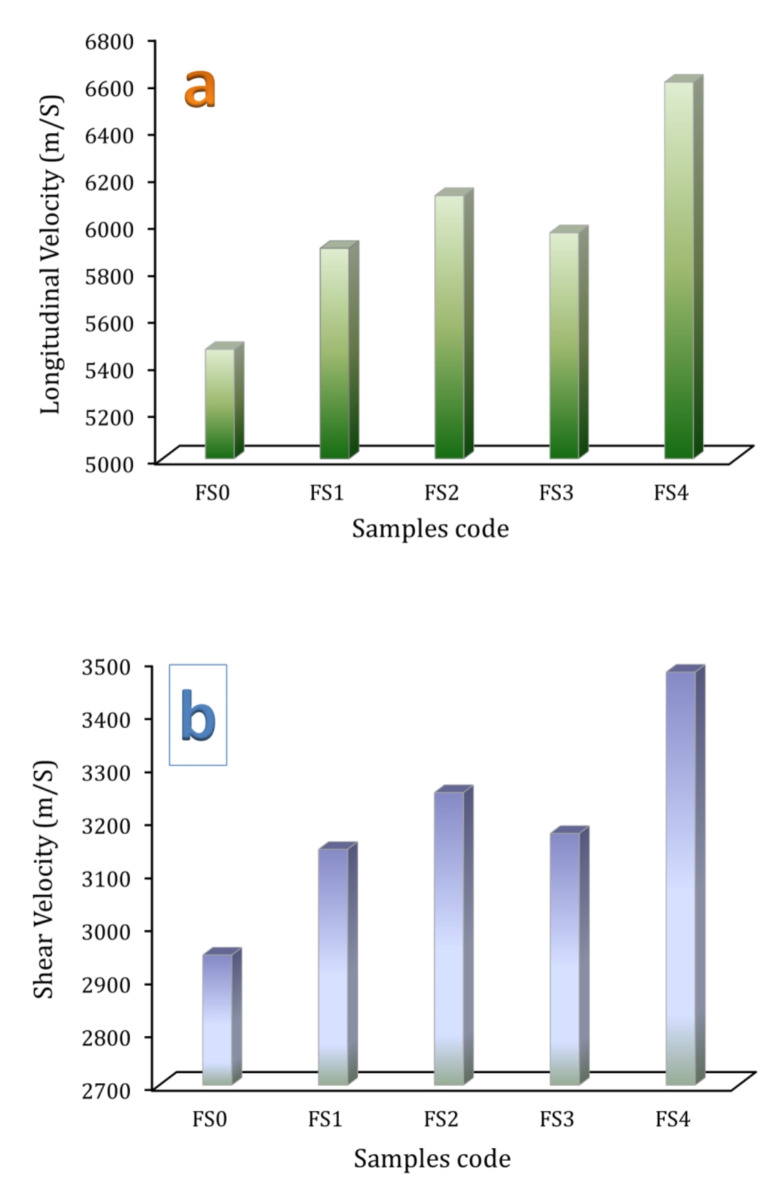
(**a**) Longitudinal and (**b**) shear ultrasonic velocities of the sintered samples.

**Fig. 12:**
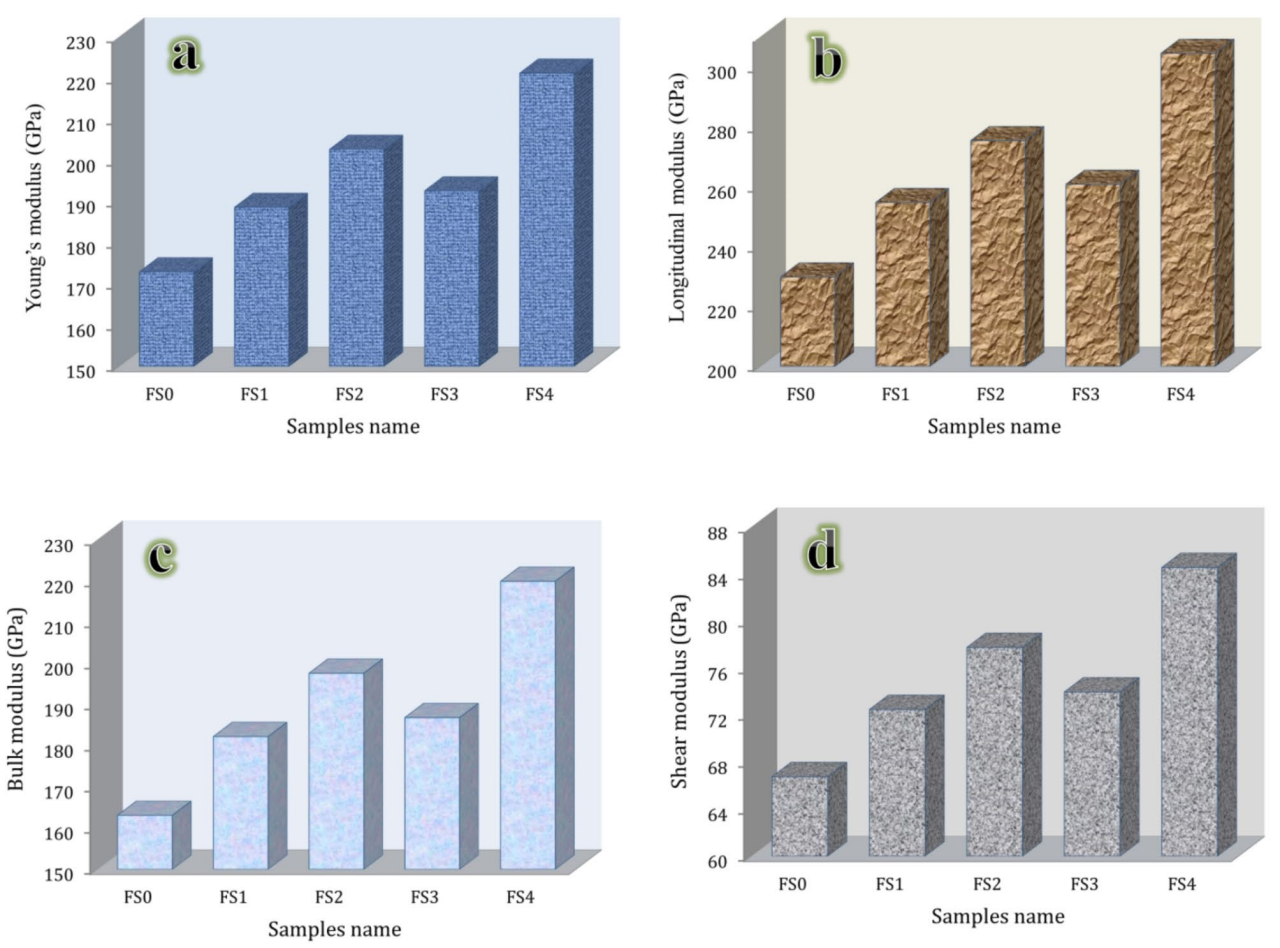
Elastic moduli of the sintered samples, i.e., (**a**) Young’s modulus, (**b**) longitudinal modulus, (**c**) bulk modulus, and (**d**) shear modulus.

Figure 11(a, b) shows the longitudinal and shear ultrasonic velocities for FS0, FS1, FS2, FS3, and FS4 samples measured by ultrasonic technique. It’s fascinating to see that more reinforcement increases ultrasonic velocities. The longitudinal velocities of previous samples are 5464.98, 58.94,25, 6118.29, 5960.25, and 6599.81 m/s, respectively, while the shear velocities of the same samples are 2945.11, 3144.28, 3251.29, 3174.25, and 3478.08 m/s, respectively. Figure 12(a-d) shows the group of elastic moduli values: longitudinal modulus, Young’s modulus, bulk modulus, shear modulus, and Poisson’s ratio of samples, respectively. The group of elastic moduli shows a similar tendency for ultrasonic velocities. For example, the longitudinal modulus of the unreinforced sample (FG0) is 229.87 GPa, which increases to 254.71, 275.38, 260.81, and 304.51 GPa for the FS1, FS2, FS3, and FS4 samples, which improves by about 10.81, 19.80, 13.46, and 32.47%, respectively. We can conclude that the nanocomposite with the most significant hybrid of reinforcements (FS4) exhibits the best mechanical properties, followed by the one with Si_3_N_4_ only (FS2), and the nanocomposite enhanced with 2.5 vol% silica fume + 2.5 vol% Si_3_N_4_ (FS3), which ranks third. This indicates that the addition of Si_3_N_4_, which has higher mechanical properties than silica fume, has a more significant effect on mechanical properties. Generally, the enhanced compressive strength and a group of elastic moduli in nanocomposite samples can be attributed to several factors.

(i) Thermal mismatch strengthening pertains to the significant disparity in the coefficients of thermal expansion (CTE) among Fe-SB base (12.51 × 10⁻⁶ /°C), Si_3_N_4_ (3.3 × 10⁻⁶ /°C), and silica fume (0.6 × 10⁻⁶ /°C) particles, which leads to the generation of thermally induced residual stresses^43^. Minor temperature fluctuations induce thermal stresses in the Fe-SB matrix, which substantially elevate dislocation density at the interface, enhancing the nanocomposite’s strength.
(ii) Due to the uniform distribution of the rigid reinforcements phase into the Fe-SB base, the Orowan strengthening effect enhances nanocomposites’ mechanical characteristics by preventing dislocation movement. Hence, dislocation loops surrounding reinforcing particles increase stress and deformation^44^.
(iii) The load transmission between the hard reinforcement particles and the Fe-SB base is contingent upon the bond’s degree^45^.

#### Tribological properties

**Fig. 13:**
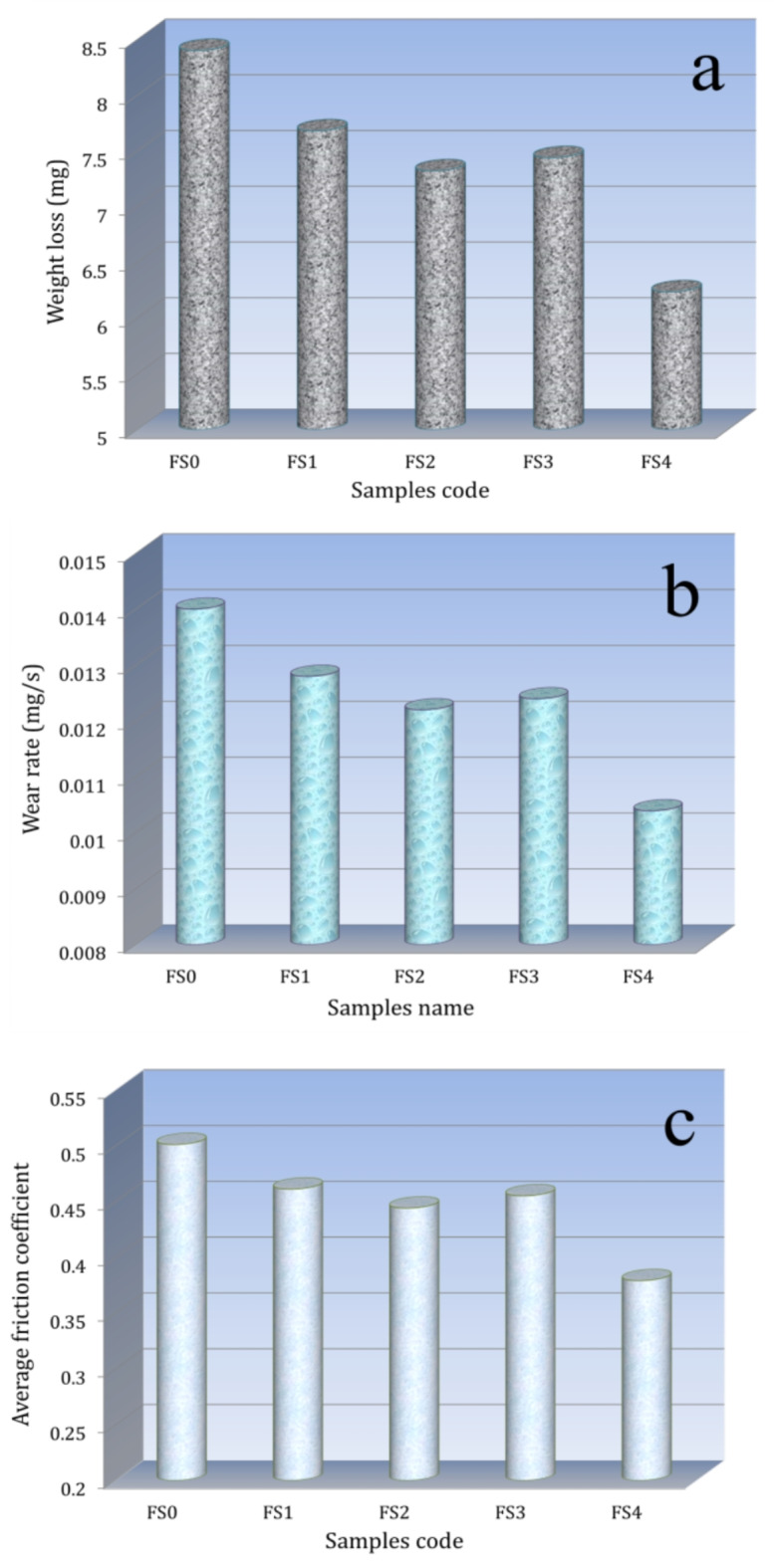
(**a**) Weight loss, (**b**) wear rate, and (**c**) fraction coefficient of the sintered samples under an applied load of 30 N.

Figure 13(a-c) represents the variations in weight loss, wear rate, and average friction coefficient of FS0, FS1, FS2, FS3, and FS4 samples under applied loads of test 30 N for 10 min. The results show that added Si3N4 and/or silica fume reinforcement positively affects the wear resistance of the Fe-SB base. A sample that isn’t reinforced (FS0) has a wear rate of 0.0140 mg/s. When mono silica fume and Si_3_N_4_ were added, the rate dropped to 0.128 and 0.0122 mg/s, 8.66 and 12.72% less than the FS0 sample. For samples containing hybrid reinforcement (i.e., FS3 and FS4), the value decreases to 0.0124 and 0.0104 mg/s, which decreases about 10.89 and 25.38% compared to the FS0 sample. The improvement in the wear rate and friction coefficient of nanocomposites can be attributed to the incorporation of Si_3_N_4_ and silica fume particles into the Fe-SB base, which enhances the microhardness and strength of the nanocomposites, consequently reducing the wear rate and friction coefficient^46^.

Moreover, the augmentation of microhardness correlates with a reduction in the actual contact area. The actual contact area is often defined by the ratio of the average load to the hardness of the pin material; hence, a reduction in the real contact area results in significant decreases in wear rate. There are previous studies related to improving the wear resistance of iron alloys by adding ceramics and their relationship to enhancing mechanical properties, including the impact of cerium oxide addition on the wear behavior of hard-faced Fe-18Cr-1.1Nb-2.1 C alloy was examined by Y K Singla et al.^47^. They discovered that the alloys containing RE significantly improved in microhardness and wore resistance, with 4 weight% being the ideal quantity of RE. According to research by Shule Xing et al.^48^, adding ceria to the Fe-Cr13-Mn-Nb hard-facing alloy enhanced its hardness and fracture toughness. This, in turn, boosted the alloy’s resistance to plastic deformation and scratches, hence improving its wear resistance. According to S.P. Sharma et al.^49^, flame-sprayed Ni-based coatings with an ideal CeO_2_ content of 0.8 weight% have an abrasive wear rate that is about 21% lower than those without CeO_2_. Grain refining and coating hardness are the reasons for the increased resistance to abrasive wear. According to M.M. Quazi et al.^50^, RE-based laser claddings have shown modest wear characteristics in most investigations.

**Fig. 14:**
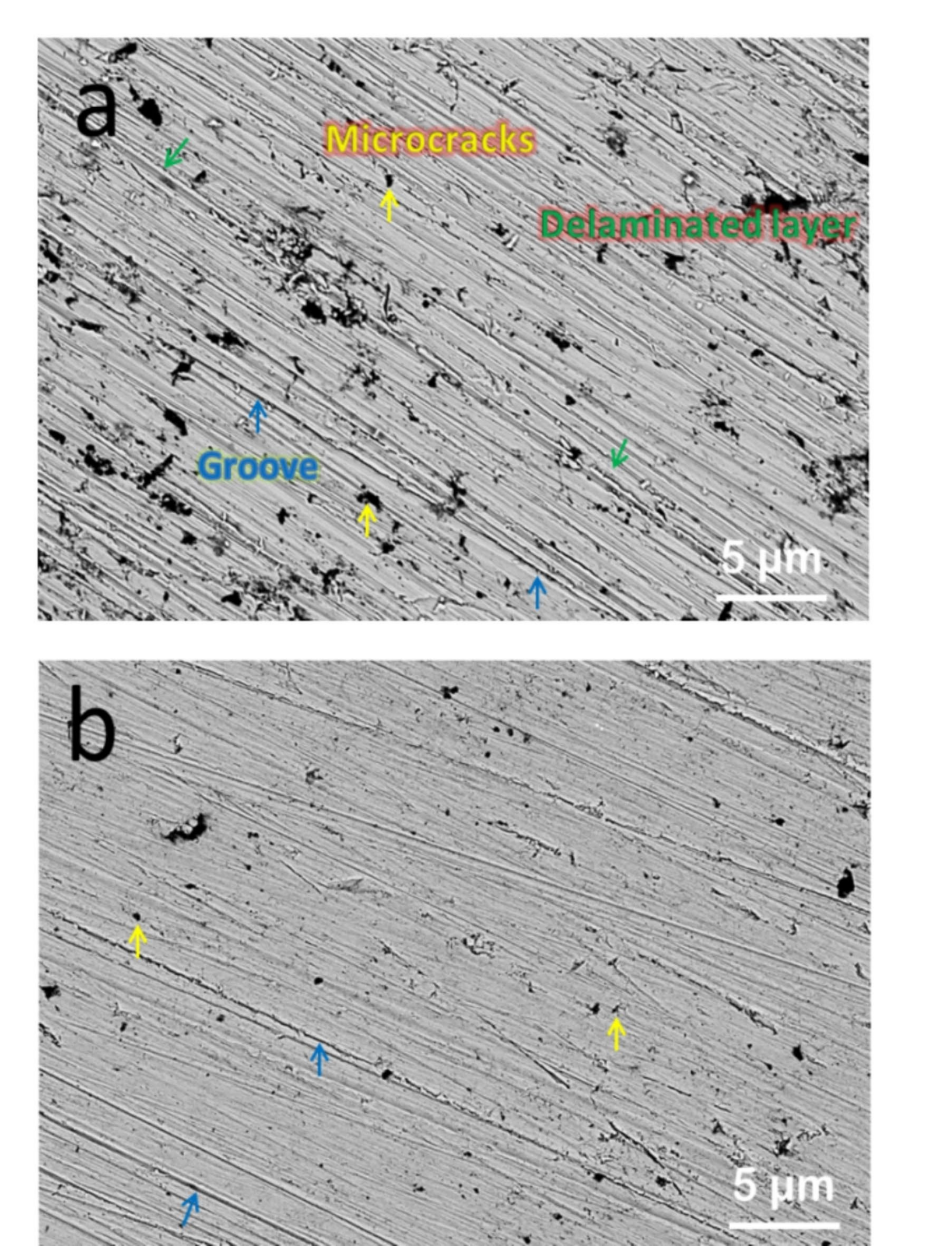
SEM images of wear tracks of (**a**) the FS0 and (**b**) the FS4 samples.

The worn surfaces of the FS0 and FS4 samples were examined under an applied stress of 30 N and a speed of 0. 8 m/s to evaluate the wear processes of the samples, as shown in Fig. 14a–b. For the base sample (FS0 sample), loose layers, and grooves appear on the wear track as shown in Fig. 14a. Surface delamination reveals adhesive wear, which includes crack initiation and propagation as well as the final fracture of the sample in the vicinity of the surface^51^. On the other hand, the samples reinforced with hybrid ceramics (FS4 sample) have a smoother surface than the base sample, and there is only sporadic debris and slight grooves on the worn surface. Some metals (Fe and SB) debris have flattened in the wear process because of its low hardness. The number of cracks appears lower in this wear track, thus, the dominant wear mechanism is abrasive wear^52^.

#### Magnetic properties

**Fig. 15:**
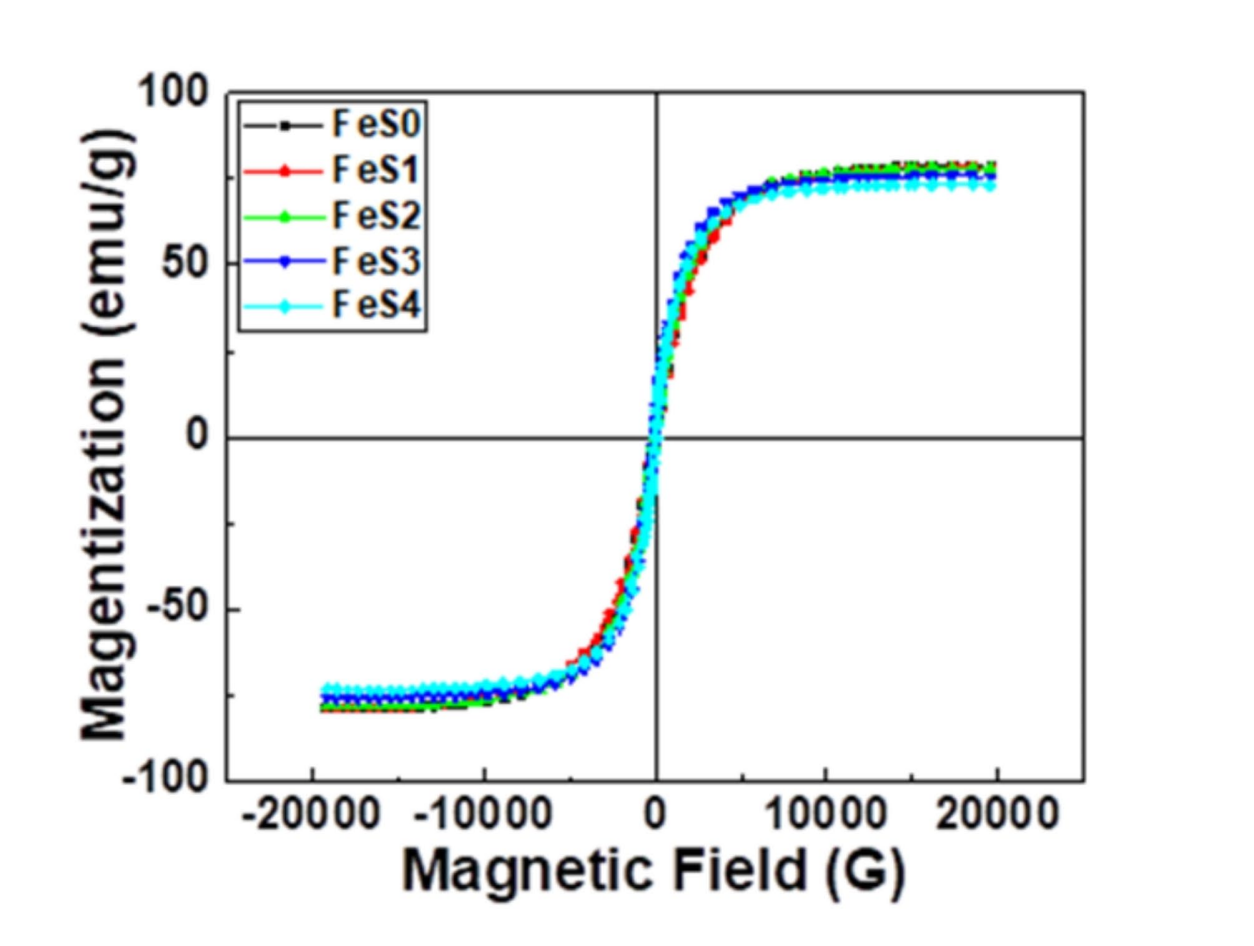
Magnetic hysteresis loops of the prepared nanocomposites.

Figure 15 shows the magnetic hysteresis loops of the FS0, FS1, FS2, FS3, and FS4 samples at room temperature. The graphs verify the ferromagnetic behavior of the samples. Table 5 provides an overview of the estimated values of the various magnetic characteristics, such as saturation magnetization (M_s_), coercivity (H_c_), and remanent magnetization (M_r_). Different Si_3_N_4_ and silica fume ratios had varying coercivity, ranging from 59.586 to 53.997 G. Their softer ferromagnetic nature is indicated by the lower coercivity values^53^. Numerous factors influence coercivity, including magnetocrystallinity, particle shape and morphology, microstrain, and size distribution^54^. The M_s_ of raw Fe-vol.%15 SiBr is roughly 78.737 emu/g, as shown in the figure, and decreases to 73.412 emu/g when 5% Si_3_N_4_ and 5% silica fume are added. Additionally, this variation in the magnetization values reflects the impact of the particle size and synthetic process on the final magnetic characteristics^55^. Based on these results, the decrease in magnetic properties is very slight on the sprout due to the noticeable improvement in mechanical properties and corrosion resistance, and this allows the use of these composites in magnetic applications.

**Table 5:** Magnetic parameters for all samples; magnetization (M_s_), Coercivity (H_C,_), and Retentivity(M_r_).

Samples name	M_S_ (emu/g)	H_C_ (G)	M_*r*_ (emu/g)
FS0	78.737	59.586	2.1659
FS1	78.455	59.146	2.0138
FS2	78.109	58.466	2.3457
FS3	75.845	55.288	2.7346
FS4	73.412	53.997	2.5476

#### Electric properties

**Fig. 16:**
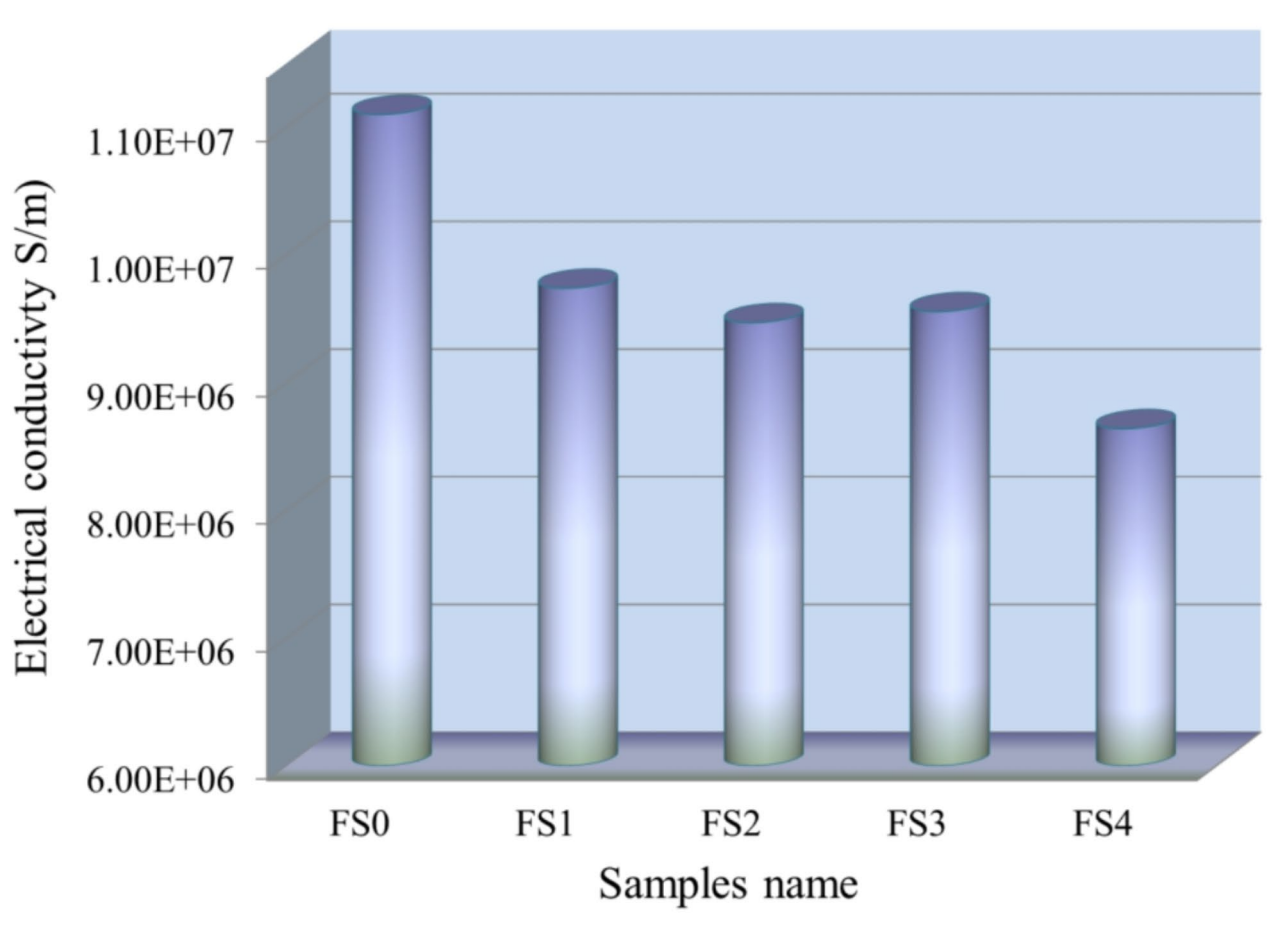
Electrical conductivity of the Fe-SiBr and its sintered nanocomposites.

The sintered nanocomposite samples’ electrical conductivity values were tested and shown in Fig. 16. As predicted, increased Si_3_N_4_ or silica fume volume percent lowers the electrical conductivity of nanocomposite samples. It is noted that the conductivity value of the Fe-Cu base was 1.110 × 10^7^ S/cm. On the other hand, after adding 5 vol% silica fume, 5 vol% Si_3_N_4_ silica, 2.5 vol% silica fume + 2.5 vol% Si_3_N_4_ and 5 vol% silica fume + 5 vol% Si_3_N_4_ reinforcements, the conductivity value decreased, recording 9.74 × 10^6^, 9.47 × 10^6^, 9.56 × 10^6^, and 8.64 × 10^6^ S/m, respectively. When discussing the findings, it is important to keep in mind that Si_3_N_4_ and silica fume (~ 10–^14^ and 10–^12^ S/m. respectively) have electrical conductivities that are much higher than those of Fe and SiBr (~ 1.04 × 10^7^ 3.6 × 10^7^ S/m, respectively), which means that they reduce electrical conductivity. Moreover, little contact between the free electrons and the nucleus defines metals, so the Fe-SiBr base has strong electrical conductivity because free electrons travel easily. Conversely, the presence of ceramic reinforcements helps strengthen the links between free electrons and the nucleus, causing an apparent reduction in the electrical conductivity of such nanocomposites^21,56,57^. It is worth noting that the addition of ceramic led to a noticeable improvement in the previous properties (mechanical properties and wear resistance), and at the same time a slight decrease in electrical conductivity, so the electrical conductivity of the prepared compounds is still a good electrical conductor.

## Conclusion

The main objectives of this work using the PM method are to reduce Fe and SiBr wastes to produce Fe 15 vol% SiBr base nanocomposites reinforced with mono and hybrid reinforcements (Si_3_N_4_ and silica fume). The following conclusions were drawn:

- The addition of ceramic positively affects the decrease in particle and crystal size, lattice strain and dislocation density, and vice versa.
- Adding Si_3_N_4_ and/or silica fume reduced the samples’ bulk and relative densities while raising their apparent porosity.
- As the Si_3_N_4_ and/or silica fume was increased, the microhardness, compressive strength, and longitudinal modulus were improved. The microhardness of FS0 to FS4 samples was 132.11, 159.14, 176.01, 166.42, and 208.74 Hv, respectively.
- Improvement of tribological properties such as wear rate and fraction coefficient) of the nanocomposites improved with added mono and hybrid reinforcement. The wear rate of FS1, FS2, FS3, and FS4 samples improved by about 8.59, 12.86, 11.49, and 25.78%, respectively, and the fraction coefficient of the same sample about enhanced 7.98, 11.38, 9.18, and 24.36%, respectively, compared with the FS0 sample.
- There was a slight decrease in the magnetization of the Fe-SiBr base (73.737 emu/g) with the addition of reinforcements, reaching 73.412 emu/g for the FS4 sample.
- Finally, the electrical conductivity of the samples decreased from 1.11 × 10^7^ S/m for the FS0 sample to 9.74 × 10^6^, 9.47 × 10^6^, 9.56 × 10^6^, 8.64 × 10^6^ with the addition of 5 vol% silica fume, 5 vol% Si_3_N_4_, 2.5 vol% silica + fume, 2.5 vol% Si_3_N_4_ and 5 vol% silica + fume, 5 vol% Si_3_N_4_, respectively.

## Data Availability

The datasets generated and/or analyzed during the current study are not publicly available because all data are presented in the article and therefore, there is no need to include raw data but they are available from the corresponding author upon reasonable request.
